# A call to create integrated services to better address the needs of migrants who use drugs in Europe

**DOI:** 10.1186/s12954-023-00923-6

**Published:** 2024-01-13

**Authors:** Lena van Selm, Trenton M. White, Camila A. Picchio, Ana Requena-Méndez, Machteld Busz, Roberto Perez Gayo, Aline Pouille, Pedro Mateu Gelabert, Jeffrey V. Lazarus

**Affiliations:** 1 Barcelona Institute for Global Health (ISGlobal), Barcelona, Spain; 2 Department of Medicine Solna, Karolinska Institute, Stockholm, Sweden; 3 Mainline, Amsterdam, The Netherlands; 4 Correlation – European Harm Reduction Network, Amsterdam, The Netherlands; 5 Department of Special Needs Education, Ghent University, Ghent, Belgium; 6 The City University of New York Graduate School of Public Health and Health Policy (CUNY SPH), New York, NY USA; 7 Faculty of Medicine and Health Sciences, University of Barcelona, Barcelona, Spain

**Keywords:** Addiction, Drug use, Harm reduction, Healthcare access, Migrant health, European Union

## Abstract

Each year, thousands of migrants enter the EU. Data on drug use in migrant populations are scarce and inconclusive. However, several risk factors make them particularly vulnerable to engaging in problematic drug use. In this perspective, we summarize the limited information that is available on migrants who use drugs and make a case as to why it is essential to improve access to health and social services, including harm reduction services, for this population. With this aim, we call for the co-creation of integrated services that better address the needs of migrants who use drugs in Europe.

## Background

Migration is now a common reality for tens of millions of people around the world. Among people who have left their country of origin to settle in another country, some portion will engage in problematic drug use [1], whether initiating in their country of origin or in the host country. Data on drug use in migrant populations are scarce and inconclusive. For example, a review identified eight studies comparing drug use in first-generation migrants to drug use in the general population of which two found higher levels in migrants, four found lower levels in migrants and two found different results depending on the type of drugs [2]. However, these studies were highly heterogeneous in their methodologies and the type of populations and drugs they included. Although, generally, migrant groups seem to have lower rates of substance use than host populations, several risk factors make them particularly vulnerable to engaging in problematic drug use [3]. Persons who migrated often face challenges in the country they settled in, including limited job opportunities and acculturation difficulties driven, in part, through poverty (as they tend to occupy lower socio-economic positions in society), language barriers, mental health problems, and the consequences of trauma [4–6]. The latter can stem from pre-migration traumatic experiences related to political conflict, war, or economic deprivation that prompted their migration, as well as the potentially traumatic migration journey itself [7–9].

Although exact numbers on the burden of drug use among migrants are inconclusive, recent global events have likely impacted the number of migrants using drugs in the European Union (EU). As a result of the war with Russia, millions of people from Ukraine, one of the countries with the highest levels of injecting drug use globally, have been entering the EU [10]. In addition, globally, migrants’ and refugees’ self-reported use of alcohol and drugs increased by 20% during the COVID-19 pandemic [11]. In March 2023, the European Monitoring Centre for Drugs and Drug Addiction (EMCDDA) recognized migration as a “megatrend”—a long-term driving force that will likely have significant future influence—in its work addressing the increasingly complex and dynamic drug markets in Europe through 2030 [12]. However, the EMCDDA European drug report 2023 only made a limited mention of drug use among migrants [13]. In summary, migrants with problematic drug use are a neglected group and should be provided with adequate support.

## Limited access to services for migrants who use drugs

Providing adequate health and social services to migrants who use drugs (MWUD) is not only a matter of human dignity and sound public health, but also of their rights under European and international law [14]. However, MWUD face significant barriers when trying to access health and social services, including in European health systems. These barriers are often rooted in a range of factors, including cultural differences such as language and (mis)understanding of the health system [15], legal status [15, 16], and discrimination [16, 17]. By denying migrants access to health and social services, irrespective of drug use, these fundamental rights are violated and perpetuate inequity, stigma, and discrimination in addition to contributing to poorer health outcomes.

## Public health benefits of improved healthcare for migrant who use drugs

Improving access to health and social services, including harm reduction services, for migrants who use drugs is crucial for promoting public health. MWUD may be at a higher risk of infectious diseases, such as HIV and viral hepatitis, due to unsafe drug use practices [18–20], poor living conditions and, in some cases, higher prevalence of these diseases in their country of origin. For example, in 2022, the prevalence of HIV in the EU increased, which has been largely attributed to refugees living with HIV that arrived from Ukraine [21]. By providing MWUD with access to harm reduction services, such as needle and syringe programs, opioid substitution therapy, and HIV and viral hepatitis B and C testing and treatment, the spread of infectious diseases will likely be reduced. Further, investment in harm reduction services is cost-effective in the promotion of public health [17, 22, 23].

## Recommendations from civil society experts on improving health and social services

MWUD may experience social marginalization or isolation due to their drug use and/or their migrant status [24]. This can result in stigma, discrimination, and exclusion from social, economic, and political opportunities. Further, unaddressed health and social needs may lead to the development or exacerbation of serious mental health conditions. Inclusive practices for treating vulnerable and marginalized groups can help improve the social and mental health of MWUD [25]. For example, including interpreters or cultural mediators in healthcare services improves the quality of care for patients [26, 27]. Civil society and health experts working with migrants who use drugs in the European Union recently published recommendations in four areas as part of an EU-funded project “Services for vulnerable migrants who use drugs in the EU (SEMID-EU)” [18] (Table 1).

**Table 1:** Areas of recommendations by civil society and health experts working with migrants who use drugs in the European Union

1. Increasing data availability and quality, to inform guidelines;	
2. Increasing the availability of drug dependency services for migrants, including screening for mental health issues and involving migrants who use drugs in the development of services;	
3. Eliminating country, and service level barriers for accessing these services, as well as providing migrants who use drugs with suitable information and combating stigma and discrimination; and	
4. The need for increased collaboration among and within EU countries regarding healthcare for migrants who use drugs, on policy level as well as service level, including civil society organizations, peer navigation and multilingual cultural mediators.”	

## Including the voices of migrants who use drugs

The civil society and health experts agreed that addiction services available in EU countries are often not sensitive to the specific needs of migrants [18]. Further, a literature search conducted in 2022 revealed no published studies on the self-reported needs of MWUD [2]. One study from Norway, later published in 2023, interviewed MWUD about their drug use and help seeking barriers; however, this study only included six participants [28]. Despite the recommendations of many international organizations to involve people who use drugs in research and program and policy development, implementation of these efforts continues to stall [29]. Evidence shows that migrant involvement has a positive impact on research, service adaptations, policy dialogues, and the social and personal circumstances of migrants when they are involved [30]. To this end, the SEMID-EU project team conducted a community-based participatory research to identify and explore the specific needs of MWUD in the EU aiming to improve availability of and access to services for this population. A report including the findings from 98 interviews with MWUD with 45 different nationalities living in Amsterdam, Athens, Berlin, or Paris provided a nuanced overview of the interrelatedness between problems these populations are facing related to their migration background, drug use, and additional factors such as homelessness. In addition, it described barriers and good practices for accessing healthcare and harm reduction services, including the differences between settings and different migrant populations [31].

## Call to co-create integrated services that better address the needs of MWUD

Improving availability of and access to health and social services for all MWUD is essential for upholding human rights, promoting public health, and facilitating social integration and is especially urgent with the current number of migrants in the EU from Ukraine. Considering that the societal vulnerability of many migrants who use drugs leads to issues on multiple life domains, such as (mental) health problems, housing, and financial issues, an integrated, holistic approach is needed that offers support across these domains. To achieve these goals, policymakers must recognize the importance of providing MWUD with access to adequate services and work to eliminate the barriers that prevent them from accessing the care they need, while engaging migrants who use drugs at every step of the process.

## Data Availability

Not applicable.

## Abbreviations

EU: European Union
MWUD: Migrations who use drugs
SEMID-EU: Services for vulnerable migrants who use drugs in the EU

## References

[1] Thanki D, Vicente J. PDU (Problem drug use) revision summary. 2013 [cited 2023 Jul 13]. https://core.ac.uk/download/pdf/89950538.pdf.

[2] van Selm L, White TM, Doran J, Pujol C, Picchio CA, Lazarus JV. Report on SErvices for vulnerable MIgrants who use Drugs in the EU (SEMID-EU). 2022. https://english.mainline.nl/posts/show/14430/services-for-vulnerable-migrants-who-use-drugs-in-the-eu.

[3] European Monitoring Centre for Drugs and Drug Addiction. Health and social responses to drug problems: a European guide. www.emcdda.europa.eu [cited 2023 Jul 4]. https://www.emcdda.europa.eu/publications/manuals/health-and-social-responses-to-drug-problems-a-european-guide_en.

[4] Horyniak D, Melo JS, Farrell RM, Ojeda VD, Strathdee SA (2016). Epidemiology of substance use among forced migrants: a global systematic review. PLoS ONE.

[5] Paradies Y, Ben J, Denson N, Elias A, Priest N, Pieterse A (2015). Racism as a determinant of health: a systematic review and meta-analysis. PLoS ONE.

[6] Savage JE, Mezuk B (2014). Psychosocial and contextual determinants of alcohol and drug use disorders in the National Latino and Asian American Study. Drug Alcohol Depend.

[7] Vignier N, Desgrées du Loû A, Pannetier J, Ravalihasy A, Gosselin A, Lert F (2018). Access to health insurance coverage among sub-Saharan African migrants living in France: results of the ANRS-PARCOURS study. PLoS ONE.

[8] Pannetier J, Lert F, Jauffret Roustide M, du Loû AD (2017). Mental health of sub-saharan african migrants: the gendered role of migration paths and transnational ties. SSM Popul Health.

[9] Kirkinis K, Pieterse AL, Agiliga A, Brownell A (2021). Racism, racial discrimination, and trauma: a systematic review of the social science literature. Ethn Health.

[10] United Nations Office on Drugs and Crime. Conflict in Ukraine: key evidence on drug demand and supply. 2022 [cited 2023 Feb 8]. https://www.unodc.org/documents/data-and-analysis/Ukraine/Ukraine_drug_demand_supply.pdf.

[11] World Health Organization. Updated recommendations on simplified service delivery and diagnostics for hepatitis C infection. 2022 [cited 2022 Dec 15]. https://www.who.int/publications/i/item/9789240052697.

[12] European Monitoring Centre for Drugs and Drug Addiction. The future of drug monitoring in Europe until 2030: A report summarising the findings and lessons learnt from the EMCDDA’s ‘futures study’. www.emcdda.europa.eu [cited 2023 Jul 4]. https://www.emcdda.europa.eu/publications/technical-reports/future-drug-monitoring-europe-until-2030_en.

[13] European Monitoring Centre for Drugs and Drug Addiction. European drug report 2023: trends and developments. 2023.

[14] Greer SL, Rozenblum S, Fahy N, Brooks E, Jarman H, Ruijter A de, et al. EU Charter of Fundamental Rights. Article 35—Health Care. In: Everything you always wanted to know about European Union health policy but were afraid to ask: Third, revised edition [Internet] [Internet]. European Observatory on Health Systems and Policies; 2022 [cited 2023 Jul 4]. https://www.ncbi.nlm.nih.gov/books/NBK590176/. 37023240

[15] De Kock C (2022). Equitable substance use treatment for migrants and ethnic minorities in Flanders, Belgium: service coordinator and expert perspectives. Subst Abuse Res Treat.

[16] Deimel D (2013). Ausländerrechtliche Rehabilitationshindernisse in der Behandlung suchtkranker Migranten. Suchttherapie.

[17] Kim SW, Pulkki-Brannstrom A-M, Skordis-Worrall J (2014). Comparing the cost effectiveness of harm reduction strategies: a case study of the Ukraine. Cost Eff Resour Alloc.

[18] van Selm L, White TM, Picchio CA, Requena-Méndez A, Busz M, Bakker I (2023). Drug use and access to drug dependency services for vulnerable migrants who use drugs in the European Union: consensus statements and recommendations from civil society experts in Europe. Int J Drug Policy.

[19] European Monitoring Centre for Drugs and Drug Addiction. Drug-related infectious diseases : health and social responses.

[20] Artenie A, Stone J, Fraser H, Stewart D, Arum C, Lim AG (2023). Incidence of HIV and hepatitis C virus among people who inject drugs, and associations with age and sex or gender: a global systematic review and meta-analysis. Lancet Gastroenterol Hepatol.

[21] European Centre for Disease Prevention and Control. HIV/AIDS surveillance in Europe 2023. 2023.

[22] Cousien A, Tran VC, Deuffic-Burban S, Jauffret-Roustide M, Mabileau G, Dhersin J-S (2018). Effectiveness and cost-effectiveness of interventions targeting harm reduction and chronic hepatitis C cascade of care in people who inject drugs: the case of France. J Viral Hepat.

[23] Wilson DP, Donald B, Shattock AJ, Wilson D, Fraser-Hurt N (2015). The cost-effectiveness of harm reduction. Int J Drug Policy.

[24] Löbel LM, Kröger H, Tibubos AN (2022). How migration status shapes susceptibility of individuals’ loneliness to social isolation. Int J Public Health.

[25] Lazarus JV, Baker L, Cascio M, Onyango D, Schatz E, Smith AC (2020). Novel health systems service design checklist to improve healthcare access for marginalised, underserved communities in Europe. BMJ Open.

[26] Heath M, Hvass AMF, Wejse CM (2023). Interpreter services and effect on healthcare—a systematic review of the impact of different types of interpreters on patient outcome. J Migr Health.

[27] Lebano A, Hamed S, Bradby H, Gil-Salmerón A, Durá-Ferrandis E, Garcés-Ferrer J (2020). Migrants’ and refugees’ health status and healthcare in Europe: a scoping literature review. BMC Public Health.

[28] Pettersen RJ, Debesay J (2023). Substance use and help-seeking barriers: a qualitative study of East African migrants’ experiences of access to Norwegian healthcare services. BMC Health Serv Res.

[29] Ti L, Tzemis D, Buxton JA (2012). Engaging people who use drugs in policy and program development: a review of the literature. Subst Abus Treat Prev Policy.

[30] MacFarlane A, Ogoro M, de Freitas C, Niranjan V, Severoni S, Waagensen E (2021). Migrants’ involvement in health policy, service development and research in the WHO European Region: a narrative review of policy and practice. Trop Med Int Health.

[31] Mainline—Services for Vulnerable Migrants who use Drugs in the EU [cited 4 Jul 2023]. https://english.mainline.nl/posts/show/14430/services-for-vulnerable-migrants-who-use-drugs-in-the-eu.

